# Boil water notices as health-risk communication: risk perceptions, efficacy, and compliance during winter storm Uri

**DOI:** 10.1038/s41598-023-50286-y

**Published:** 2024-01-08

**Authors:** Ashleigh M. Day, Sydney O’Shay, Khairul Islam, Matthew W. Seeger, F. Gianluca Sperone, Shawn P. McElmurry

**Affiliations:** 1 School of Communication, Northern Arizona University, Flagstaff, USA; 2 Department Communication Studies and Philosophy, Utah State University, Logan, USA; 3 Department of Communication Studies, State University of New York at Oswego, Oswego, USA; 4 Department of Communication, Wayne State University, Detroit, USA; 5 Department of Environmental Science and Geology, Wayne State University, Detroit, USA; 6 Department of Civil and Environmental Engineering, Wayne State University, Detroit, USA

**Keywords:** Environmental social sciences, Natural hazards

## Abstract

Winter Storm Uri was a disaster that impacted much of the United States during February of 2021. During and after the storm, Texas and Oklahoma experienced massive power grid failures. This led to cascading impacts, including water system disruptions and many boil water notices (BWNs). The breakdown of some communication channels and the inability to enact protective actions due to power outages, as well as travel limitations on public roads, complicated the dissemination and implementation of notifications. This research examined individuals’ perceptions of risk, water quality, and BWNs during Uri. Additionally, this study sought to understand if previous experience with a BWN influenced compliance during Uri and how perceived efficacy impacted these variables. Surveying 893 Texans and Oklahomans revealed that most Uri-affected respondents believed the risks associated with BWNs were severe. Income and race were two factors that influenced BWN compliance. Age, gender, and level of education did not influence compliance. Previous experience with BWNs did not increase risk perceptions. Higher levels of perceived efficacy correlated to higher levels of compliance, perceptions of risk, and water quality, much of which support propositions of the Extended Parallel Process Model. Results suggest that pre-disaster planning and communication are imperative to helping reduce risk(s) and enhancing efficacy *during* a disaster, especially for novel disasters that have cascading risks, like Winter Storm Uri.

## Introduction

Winter Storm Uri occurred February 12–16, 2021. Uri was a coast-to-coast storm system that produced record-amounts of snow and damaging ice and caused many other weather-related issues^1^. In several regions across Texas, Uri dropped 4–6 inches of snow. The storm system produced the coldest temperatures on record for most Texas cities. Nearby Oklahoma was also severely impacted. Oklahoma City reported 6 inches of snow and drifts of 2–4 feet at the Will Rogers Airport^1,2^. In addition to snow, ice, and wind, Uri also impacted power grids and water systems.

Uri caused wide-spread power outages for over 9.7 million people in the United States and Mexico^2^. Due to power failures, water treatment plants across Texas failed to keep water moving, pipes froze, and there were many pipe leaks^3,4^. Nearly 15 million Texans experienced disruptions to their primary source of potable water^2,5^. Four days after the beginning of Winter Storm Uri, February 16, 2021, 1.4 million Texans still lacked reliable drinking water service. More than 200,000 Texans were still without water on February 25 as snow, ice, and freezing temperatures persisted. Due to the potential of contaminated drinking water, boil water notices (BWNs) were issued across Texas and Oklahoma. According to data obtained from the Texas Commission of Environmental Quality^4^, there were 1,105 BWNs that impacted about 14.5 million Texans from February 15–19, 2021. Overall, almost 40% (1985) of public and community water systems in Texas had a BWN during Winter Storm Uri^6^. However, it is likely that many people under a BWN did not receive timely notification about water contamination risks.

### BWNs as risk communication

BWNs, like those issued during Uri, are *risk* messages^7,8^. However, crisis communication generally warns individuals about a current threat, risk messages warn individuals about a potential threat that *could* occur in the future^9–12^. Since BWNs aim to inform affected populations about potential water contamination and advise them to take protective actions, they can be classified as risk messages. The Extended Parallel Process Model (EPPM) suggests efficacy plays a key role in determining whether individuals enact protective behaviors^13^. EPPM asserts the effectiveness of crisis and risk communication depends, in part, on conveying efficacy (response + self) information *and* informing publics about risks in a way that incites action versus fear control^13,14^. Further, at-risk individuals tend to view ‘perceived effectiveness in protecting health’ during a water contamination emergency as “the most important correlate of protective action,” which implies that risk communicators must clearly explain how *and* why a protective action will protect an individual’s health (p. 887)^14^. However, it is not clear if this communication strategy also applies in a novel disaster context that has many cascading risks, like Winter Storm Uri.

Cascading risks complicate communication during disasters. Cascading risks are largely associated with “the anthropogenic domain and the vulnerability component of risk. This results in a disaster escalation process. In other words, it focuses mainly on the management of social and infrastructure nodes” (p. 2253)^15^. Simply, cascading risk(s) occur when one hazardous event (e.g., Winter Storm Uri) triggers other events/risks, which produce even more severe consequences (e.g., BWNs, infrastructure failure). Thus, effective communication is key to protecting health and safety when a disaster has cascading risks. More particularly, clear communication of BWNs can enhance individuals’ efficacy levels by persuading them to properly boil their tap water, or use another suitable water source (e.g., bottled water) to mitigate contamination risks, especially during a disaster with cascading risks like Winter Storm Uri^8,15–17^.

To create effective risk messages, communicators must understand risk perception and how it influences actions of at-risk individuals. Simply, risk perception is “the belief that one is vulnerable” to some future risk such as disease, floods, etc.^18^. While risk perception can be conceptualized in many ways, Rimal and Real^18^ view risk perception as a combination of a person’s *perceived susceptibility* and *perceived severity* of a risk and these are assessed based on threats that may occur in the *future*. While risk perception alone is typically not enough to predict behavioral intentions, it does help indicate the communicative needs of at-risk individuals and it can help guide message development^8,12,18–20^. Risk perception is an integral element for risk communication as it helps communicators align their message(s) with audience (i.e., at-risk individuals) concerns and needs^10,12,19^. As may be evident, there is a gap in research related to investigating these phenomena, specifically, as related to BWNs and risk communication. As such, investigating risk perception in the current study is important as it has important implications for risk communication in the context of disasters with cascading risks, like BWNs resulting from Winter Storm Uri.

Winter Storm Uri was a disaster with various cascading risks, with one such risk being possible water contamination. The lack of electricity due to the winter storm made communicating with at-risk individuals challenging. Previous research about BWNs notes that “the quick response of utility managers is important to protect more consumers, and using the news media as the only means to protect consumers may not provide high levels of public health protection,” (p. 2051)^21^. Not only does the context of Uri exemplify an extremely complex disaster, but it also highlights the communication difficulties that utility managers, city and state governments, and at-risk individuals faced when ‘normal’ communication channels—such as news media and social media—were not fully available or able to reach all at-risk populations due to limitations related to travel, electrical and cellphone service, etc.^1–5^.

As such, the goal of this study was to investigate BWN-affected individuals’ risk perceptions, water quality perceptions, and perceived efficacy as related to compliance with BWNs during a complex disaster with cascading risks: Winter Storm Uri. Additionally, the aforementioned phenomena can reveal communicative exigencies of affected individuals as well as help inform recommendations for future risk communication about BWNs that emerge from larger disasters.

### Current study

There is limited risk communication research focused on drinking water issues, especially during severe weather events, which often involve cascading risk(s)^15,22^. Despite awareness of an active BWN, compliance with suggested protective actions varies among affected populations. BWN compliance rates are reported to range from 36 to 98%^23^. Previous experience with a risk may increase risk perception and encourage positive protective actions^12,19,24–26^. Further, “Past experience with a hazard is generally thought to influence one’s recognition that a risk exists and increases motivation to protect one-self,” (p. 1841)^27^. As such, the following hypothesis was employed to investigate the relationship between past experience with a BWN and risk perception during Winter Storm Uri:

#### H1

Previous experience with a BWN(s) will alter individuals' perceptions of water quality and the risk of acquiring a waterborne disease during Uri.

Due to the complex nature of Winter Storm Uri and its cascading risks, communicating with affected individuals was difficult. For instance, one study reported that more than half of Uri-affected respondents were unsure or confused about whether they were under a BWN during Uri^22^, highlighting a severe risk communication issue. This finding implies serious communication gaps between officials/utility companies and the public. Perhaps even more concerning is that extant research denotes that racial minority communities (e.g., Black and Latinx communities) and lower-income household tend to have less access to clean, safe water^28^. More specifically, during Uri, racial minorities and lower-income households experienced more severe issues, like burst pipes^29^. On the other hand, communities with larger populations of non-Hispanic White residents, single family homes, and higher-income households experienced a smaller percentage of power outages after the storm^30^, meaning they likely had access to electricity, communication technologies, and running water sooner than other communities with higher populations of racial minorities and lower-income households. This information further complicates risk communication surrounding Uri, especially related to BWNs, but also highlights the need for better risk communication that is specifically tailored to diverse populations’ communicative needs.

Another factor to consider when crafting risk communication for BWNs is increasing compliance among various at-risk populations. Common reasons for non-compliance during a BWN include forgetfulness, perceived inconvenience, appearance of clean water, not believing the initial notification of a BWN, and failing to receive communication about an issued BWN^22,29,31,32^. Furthermore, low risk perception is consistently reported as a primary reason for non-compliance^31–33^. However, Americans have consistently expressed concerns about contamination of drinking water with 83% reporting a “great deal” or “fair” amount of worry in 2022^34^. Perceptions about the quality and safety of water may interact with risk perceptions and efficacy beliefs to impact decisions about protective actions^13,35,36^. Both self- and response-efficacy are important in relation to compliance with recommended protective actions during disasters. Efficacy plays a role in individuals’ perceived risk^18^, which is important to know when creating risk communication. However, there were vast communication issues surrounding Winter Storm Uri. The interactive role of risk perception and efficacy^13,35,36^ and compliance in the context of BWN’s following winter storms is not well understood. Understanding these phenomena could contribute to more effective risk communication practices during complex disasters, such as Uri. Therefore, EPPM^13^ and extant research suggesting that risk perception and efficacy beliefs play a role in people’s decisions to take protective actions (or not) when experiencing potential water contamination, inform the following hypothesis:

#### H2

Higher levels of perceived efficacy will be related to individuals’ (risk) perceptions of water quality and the risk of acquiring a waterborne disease during Uri.

#### H3

General risk perception(s), efficacy, and (risk) perception of water quality will influence compliance with BWNs during Uri.

A more detailed discussion of literature on EPPM, risk and crisis communication, and methods are provided in Supplemental Information (SI). To address gaps in understanding how risk perceptions influence response to BWNs surrounding a weather-related crisis, the 2021 Winter Storm Uri, and to evaluate our hypotheses, we conducted a survey of Texas and Oklahoma residents aimed at understanding how individuals perceive risk surrounding BWNs associated with Uri.

## Results

### Preliminary analysis

Some 99.9% of respondents reported they were affected by the storm from February 14 to February 26^7^. More than half (53.2%) of the respondents reported that they had no running water, 58% had no electricity, 75% had low water pressure, 28% had discolored water, 21% reported water with a bad smell, and 31% had a frozen water pipe. The majority (83.2%) of the respondents received BWNs or related advisories.

### Descriptive statistics

Overall, results show that perceptions of risks surrounding BWNs during Uri were high, with means score was 3.5 on a five-point scale (all reported results are based on a five-point scale, unless noted otherwise). Perceived severity of the risk associated with BWNs saw a higher mean (*M* = 4.29, *SD* = 0.72) than susceptibility (*M* = 2.41, *SD* = 1.50). Respondents reported overall satisfaction with household water during normal conditions (median response = somewhat satisfied, *M* = 3.81, *SD* = 1.22), perceived it as safe (median response = somewhat safe, *M* = 4.09, *SD* = 1.11), and rated the quality as higher than average (median response = good, *M* = 2.86 out of 4, *SD* = 0.82).

### Testing hypothesis 1 (H1)

A significant difference was observed in respondents’ perception of water quality based on whether they had previous experience with BWNs (U = 71,076.0, z = − 2.174, n = 791, *p *= 0.030). No significant difference was detected in perceived risk of acquiring a waterborne disease based on previous experience with BWNs (U = 66,877.5, z =  − 0.052, n = 733, *p *= 0.959). However, a difference in risk perception was observed between respondents that were under a BWN and those that were not (U = 23,453.5, z =  − 2.932, n = 707, *p *= 0.003), with those under a BWN scoring higher on the risk perception scale (median = 3.5 vs. 3.0). A similar difference was not observed in perceived water quality between these groups that were and were not under a BWN (*p *= 0.204). Nonetheless, even when restricting the analysis to only respondents that report being under a BWN, the difference in perceived water quality held up (U = 47,841.0, z =  − 2.664, n = 662, *p *= 0.008) and no difference was detected in perceived risk (U = 46,032.5, z =  − 0.283, n = 613, *p *= 0.777). These results offer partial support for H1, that previous experience with a BWN(s) alters an individuals' perceptions of water quality and risk of acquiring a waterborne disease during Uri.

### Testing hypothesis 2 (H2)

Small but significant correlations were observed between the levels of perceived efficacy and water quality and risk perceptions (Table 1). Efficacy had a small but significant correlation with the perceived risk of acquiring a waterborne disease during Uri (${\tau }_{B}$=0.055, n = 723, *p *= 0.043). Efficacy also correlated with water quality perception during Uri (${\tau }_{B}$=0.200, n = 760, *p *< 0.0005). Both relationships provide evidence supporting H2. Family income was found to correlate, sometimes inversely, with all perceptions investigated (Table 1).

**Table 1:** Perception and demographic relationships measured by Kendell’s tau-b (${\tau }_{b}$).

	Risk	Efficacy	Water quality	Susceptibility	Severity
Efficacy					
Correlation coef. (${\tau }_{b}$)	0.055*				
Sig. (2-tailed)	0.043				
N	723				
Water quality					
Correlation coef. (${\tau }_{b}$)	− 0.194**	0.200**			
Sig. (2-tailed)	< 0.0005	< 0.0005			
N	733	760			
Gender					
Correlation coef. (${\tau }_{b}$)	0.079*	− 0.039	− 0.059	0.066*	0.046
Sig. (2-tailed)	0.014	0.203	0.056	0.043	0.162
N	720	747	775	728	747
Age					
Correlation coef. (${\tau }_{b}$)	0.074*	0.027	0.070*	0.075*	0.040
Sig. (2-tailed)	0.015	0.365	0.016	0.015	0.207
N	719	746	774	727	746
Family income					
Correlation coef. (${\tau }_{b}$)	− 0.139**	0.062*	0.179**	− 0.159**	− 0.009
Sig. (2-tailed)	< 0.0005	0.039	< 0.0005	< 0.0005	0.768
N	637	657	685	643	659
Education					
Correlation coef. (${\tau }_{b}$)	− 0.040	0.055	0.119**	− 0.100**	0.074*
Sig. (2-tailed)	0.207	0.076	< 0.0005	0.002	0.025
N	720	746	774	727	747

*N* Sample size.

Significant values are in bold.

### Testing hypothesis 3 (H3)

Several binary logistic regression models were investigated to evaluate H3 (Table 2). Respondents’ ability to boil water was always a significant variable (*p *< 0.0005). When respondents reported they had some ability to boil water, they were more than 4.6 times more likely to comply with the BWN (OR = 4.63–4.70). Interestingly, when respondents reported not having any limitation to boil water (i.e., full ability to boil water), the likelihood was slightly less (OR = 2.73–2.89, *p* $$\le$$ 0.006). Family income and race were often significant as well, although the level of significance was not consistent (*p *= 0.019–0.064). Nonetheless, respondents who reported a family income greater than $35,000 were twice as likely to boil water (OR = 1.96–3.49) compared to those making less than $35,000. Race was at least moderately significant (*p* $$\le$$ 0.07) in all models. While there were 35 respondents who reported two or more races, the number of non-white respondents was small (n $$\le$$ 10), limiting the statistical power of our analysis. Nonetheless, across all models, American Indian or Alaskan Native respondents were about 80% less likely to report boiling water than respondents who reported to be White (*p* $$\le$$ 0.010). Age, sex, and level of education of respondents were not found to be significant predictors of BWN adherence (*p *> 0.1). Therefore, models with these variables were not included in Table 2.

**Table 2:** Logistic regression model results evaluating the influence of consumer perceptions of risk, water quality, and efficacy on whether respondents reported boiling water.

	Model 1		Model 2		Model 3	
** − **2 log likelihood	388.236		406.747		386.301a	
r^2^ (Nagelkerke)	0.196		0.211		0.202	
Chi-squared test (χ^2^)	61.496		70.119		63.431	
Degrees of freedom (df)	13		13		14	
*p* value	< 0.0005		< 0.0005		< 0.0005	
Sample Size (N)	492		514		492	

Predictor variables	OR (95% CI)	*p* value	OR (95% CI)	*p* value	OR (95% CI)	*p* value
Constant	0.13	0.064	0.05	0.001	0.07 (0,0)	0.028
Risk perception	0.87 (0.57,1.31)	0.489			0.93 (0.61,1.43)	0.750
Water quality perception			1.28 (0.99,1.64)	0.055	1.21 (0.93,1.58)	0.162
Perceived efficacy	1.75 (1.10,2.80)	0.019	1.56 (0.97,2.5)	0.064	1.59 (0.97,2.59)	0.065
Minor in household	1.7 (0.96,2.99)	0.068	1.98 (1.13,3.46)	0.017	1.73 (0.98,3.05)	0.058
Ability to boil water—none		< 0.0005		< 0.0005		< 0.0005
Ability to boil water—likely some	4.63 (2.43,8.85)	< 0.0005	4.70 (2.5,8.85)	< 0.0005	4.63 (2.42,8.86)	< 0.0005
Ability to boil water—full	2.89 (1.38,6.05)	0.005	2.73 (1.34,5.59)	0.006	2.88 (1.37,6.04)	0.005
Family income (less than $35,00)		0.019		0.052		0.030
Family income ($35,000–$49,999)	3.04 (1.17,7.94)	0.023	2.74 (1.08,6.95)	0.034	2.89 (1.11,7.56)	0.030
Family income ($50,000–$74,999)	3.49 (1.56,7.84)	0.002	2.94 (1.35,6.43)	0.007	3.37 (1.50,7.57)	0.003
Family income (75,000–$99,999)	2.64 (1.17,5.99)	0.020	2.42 (1.08,5.38)	0.031	2.50 (1.10,5.69)	0.029
Family income ($100,000 or more)	2.20 (1.08,4.46)	0.029	1.96 (0.96,3.99)	0.063	2.00 (0.97,4.10)	0.060
Race (white)		0.070		0.037		0.064
Race (Black or African American)	0.37 (0.07,2.03)	0.250	0.41 (0.08,2.15)	0.288	0.37 (0.07,2.07)	0.258
Race (American Indian or Alaska Native)	0.17 (0.04,0.66)	0.010	0.14 (0.04,0.54)	0.004	0.16 (0.04,0.65)	0.010
Race (Asian)	201,025,364	0.999	173,193,976	0.999	178,112,581	0.999
Race (two or more races)	1.69 (0.52,5.47)	0.379	1.66 (0.56,4.86)	0.359	1.84 (0.57,5.99)	0.308

*OR* Odds ratio, *CI* Confidence interval.

The first model (Table 2, Model 1) evaluated the likelihood that respondents boiled water based on perceptions of risk and efficacy as well as respondents’ ability to boil water, as defined by Day et al. and the American Water Works Association (AWWA)^7,37^ and whether a minor was in the household. While the model explained a small percentage of the variability in response (r^2^ = 0.196), it was significant (χ^2^ = 61.496, *p *< 0.0005). Risk perception, as defined by Rimal and Real^18^, had an insignificant (*p *= 0.489) inverse relationship with the likelihood respondents reported boiling water. Perceived efficacy (OR = 1.75, *p *= 0.019) and ability to boil water (*p *< 0.0005) were positively associated with adherence to BWNs. The presence of a minor in the household increased the likelihood that survey respondents reported boiling their water by 70%, although this relationship was moderately significant in this model (*p *= 0.068).

The next model (Table 2, Model 2) evaluated the same parameters as Model 1 but replaced the risk perception variable with the reported perception of water quality. This model behaved similarly overall (r^2^ = 0.211, χ^2^ = 70.119, *p *< 0.0005). Respondents’ perception of water quality was positively associated with the likelihood of boiling water (OR = 1.28), although this was only marginally significant (*p *= 0.055). For each unit increase in water quality scale, there is approximately a 28% increase in the likelihood water will be boiled before consumption. Like the previous model, perceived efficacy (OR = 1.56, *p *= 0.033) and ability to boil water (*p *< 0.0005) positively influenced BWN adherence. The presence of a minor in the household nearly doubled the likelihood that water was boiled (OR = 1.98, *p *= 0.017).

In Model 3, we included both perceptions of risk and water quality. Similar to Model 1, risk perception was not a significant predictor (*p *= 0.750). Similar to Model 2, the perception of water quality was again positively associated with whether respondents boiled water, although this was not significant (*p *= 0.162). The influence of respondents perceived efficacy (OR = 1.59, *p *= 0.65) and whether a minor was in the household (OR = 1.73, *p *= 0.058) observed in this model was similar to results observed for Models 1 and 2.

Because the risk perception scale was insignificant in all of the binary logistic regression models investigated, we also explored the influence of the susceptibility and severity scales that constituted the risk perception scale defined by Rimal and Real^18^. In this set of models (Table 3), predictor variables that were at least moderately significant in previous models were included. Water quality perception (OR = 1.16–1.19, *p *= 0.215–0.266), perceived efficacy (OR = 1.45–1.70, *p *= 0.025 to 0.145), whether a minor was in the household (OR = 1.75–1.80, *p *= 0.042 to 0.054), the ability to boil water (*p *< 0.0005), family income (*p *= 0.020–0.028) and race (*p *= 0.63–0.074) behaved similarly across all models and was consistent with Models 1–3. The focus of Models 4–6, measures of susceptibility and severity, were not found to be significant predictors with *p* values greater than 0.122 in all cases.

**Table 3:** Logistic regression model results evaluating the influence of susceptibility and severity scales that constituted the risk perception scale on whether respondents reported boiling water.

	Model 4		Model 5		Model 6	
** − **2 log likelihood	383.759		386.528		387.756	
r^2^ (Nagelkerke)	0.209		0.205		0.201	
Chi-squared test (χ^2^)	65.973		64.695		63.467	
Degrees of freedom (df)	15		14		13	
*p* value	< 0.0005		< 0.0005		< 0.0005	
Sample size (N)	492		496		496	

Predictor variables	OR (95% CI)	*p* value	OR (95% CI)	*p* value	OR (95% CI)	*p* value
Constant	0.12	0.069	0.22	0.143	0.33	0.250
Susceptibility	0.83 (0.62,1.10)	0.197	0.84 (0.64,1.11)	0.226	0.81 (0.62,1.06)	0.122
Severity	1.23 (0.86,1.77)	0.258				
Water quality perception	1.19 (0.91,1.55)	0.215	1.16 (0.89,1.52)	0.266		
Perceived efficacy	1.45 (0.88,2.39)	0.145	1.59 (0.98,2.58)	0.059	1.70 (1.07,2.71)	0.025
Minor in household	1.75 (0.99,3.09)	0.054	1.80 (1.02,3.18)	0.042	1.77 (1.00,3.12)	0.049
Ability to boil water—none		< 0.0005		< 0.0005		< 0.0005
Ability to boil water—likely some	5.05 (2.6,9.77)	< 0.0005	4.74 (2.48,9.07)	< 0.0005	4.79 (2.50,9.14)	< 0.0005
Ability to boil water—full	3.03 (1.44,6.38)	0.004	2.84 (1.36,5.93)	0.006	2.87 (1.37,5.99)	0.005
Family income (Less than $35,00)		0.027		0.028		0.020
Family income ($35,000–$49,999)	1.45 (0.83,2.55)	0.194	1.47 (0.84,2.58)	0.174	1.54 (0.89,2.68)	0.126
Family income ($50,000–$74,999)	0.43 (0.24,0.77)	0.005	0.44 (0.25,0.78)	0.005	0.44 (0.25,0.78)	0.005
Family income (75,000–99,999)	1.41 (0.71,2.79)	0.326	1.37 (0.69,2.69)	0.369	1.40 (0.71,2.76)	0.33
Family income ($100,000 or more)	0.95 (0.49,1.86)	0.882	0.98 (0.50,1.90)	0.945	0.97 (0.5,1.87)	0.915
Race (White)		0.074		0.063		0.069
Race (Black or African American)	0.41 (0.07,2.32)	0.312	0.39 (0.07,2.09)	0.271	0.39 (0.07,2.07)	0.267
Race (American Indian or Alaska Native)	0.16 (0.04,0.66)	0.011	0.16 (0.04,0.64)	0.010	0.16 (0.04,0.64)	0.010
Race (Asian)	191,763,424	0.999	178,532,594	0.999	199,567,944	0.999
Race (two or more races)	1.85 (0.56,6.18)	0.315	1.83 (0.56,6.01)	0.320	1.71 (0.52,5.6)	0.377

*OR* Odds ratio, *CI* Confidence interval.

## Discussion

The Winter Storm Uri produced various cascading risks for individuals living in Texas and Oklahoma. Findings from this research highlight, (a) that most Uri-affected respondents believed the water risks were severe, (b) that some demographic variables impacted BWN compliance, while previous BWN experiences decreased water quality perceptions but *did not* increase risk perceptions, implying a possible risk paradox effect^25^, (c) that higher levels of perceived efficacy correlated to higher levels of BWN compliance, and (d) risk perception^18^ had an inverse relationship to respondents’ boiling their water. These results highlight the need for effective risk communication during these types of disasters, as it could be the difference in compliance with protective actions^12,38^. As noted previously, non-compliance during a BWN can be a result of forgetfulness, perceived inconvenience, appearance of clean water, not believing the initial notification of a BWN, and/or failing to receive communication about an issued BWN^22,29,31,32^. Additionally, low risk perception is consistently reported as a primary reason for non-compliance with BWNs^31–33^. As suggested by our results, a lack of clear communication, perceived inconvenience, inability to boil water, and, at times, influence from risk perception impacted respondents’ BWN compliance. However, communicating BWNs was complicated during Uri.

More than half (58%) of respondents reported that they had lost electricity, complicating their access to BWN messages. Yet, the majority (83.2%) of the respondents did report receiving BWNs or related advisories *at some point during Uri or soon after Uri.* A small number of respondents (3.6%) reported that they were unsure whether they were under a BWN^7^. These findings have important implications for theory, practice, and future inquiry. Moreover, results from this research can inform future risk communication praxis in the context of BWNs^7,12,38^. Understanding risk perception, water quality perceptions, and perceived efficacy (in relation to BWN compliance) are important elements to understanding the communicative needs among at-risk individuals. These phenomena are important for developing effective and tailored risk communication^10,12,38^.

Results indicate that most respondents believed the water risks associated with Uri were severe and, thus, many had high risk perception about these events. However, previous experience with BWNs did not significantly influence risk perceptions, but perceived efficacy did show a (small) significant correlate with individuals’ perceived risk of acquiring a waterborne disease. These results can be partially explained by risk paradox^25^, which has significant implications for risk communication. It is often assumed that if an individual has high risk perception about a threat, they will be more likely to prepare and/or enact risk mitigation behavior(s); however, the opposite can also occur^25^. Sometimes, individuals with high-risk perception and/or previous experience with a particular risk still do not adequately prepare for future risks, for a variety of reasons. First, individuals may understand the risk posed by BWNs, but choose to accept the risk, perhaps because they are overburdened by other risks, such as those caused by Uri (e.g., lack of electricity, inability to travel to get supplies)^7,25,38^. Second, individuals may understand the risk posed by BWNs, but they may see someone else as responsibility for enacting the protective action (e.g., boiling water), such as the head-of-the-house, a spouse, a parent, etc.^25^. Third, individuals may understand the risks and would like to enact the protective action (e.g., boil water), but they may lack the resources to do so^25^. For instance, during Uri, many individuals did not have electricity, which, in many cases, hindered their ability to boil water. Furthermore, travel conditions were not safe during Uri. Texas and Oklahoma generally lacked the infrastructure to clear roadways in a safe and timely manner^1–5^. Thus, developing risk communication in the context of Winter Storm Uri was complicated.

There has been limited research examining BWNs that occur due to weather-related disasters^7,23^. Severe weather events, such as Uri, can create cascading risks, leading to additional challenges with communication^15,39^. Thus, these findings contribute new knowledge about a specific form and context for crisis and risk communication. Results are supportive of EPPM propositions as efficacy is positively correlated with increased risk mitigation behavior (i.e., boiling water). Additionally, perceived efficacy had a positive relationship with perceptions of risk and water quality. Some of our results, therefore, are consistent with extant research that has used EPPM in other contexts, such as in a hypothetical weather-related emergency and a radiological “dirty” bomb event^40^, hearing-loss protection for agricultural workers^41^, smokers’ risks and readiness to quit^42^, and colorectal cancer screenings^43^. However, the nature of Winter Storm Uri presents important, contextual factors that require further inquiry.

According to EPPM, exposure to a fear-appeal message prompts an individual to appraise the threat and then appraise their ability to prevent the threat/comply with protective actions^13,35^. However, perceptions *of BWNs during an extreme weather disaster like Uri* may fundamentally differ from BWNs that occur outside of a weather disaster. BWNs during extreme weather disasters may be one of many risks and risk messages that individuals are receiving and appraising. Stated another way, risk communication within the larger context of a crisis may differ from risk communication in normal times. During ‘regular’ BWNs, individuals may feel confident in their ability to comply, but during an extreme event like Uri, individuals’ ‘regular’ perceived efficacy and sense of threat may be altered since Uri disrupted power, made roads impassable, and created other risks. In many cases individuals may have been unable to comply with the BWN. Thus, in this context, BWNs could have been considered a ‘dread risk’ since they were one of numerous cascading risks^19,24^.

Dread risk accounts for whether a given threat is perceived as very severe, controllable, catastrophic, fatal, increasing, involuntary, and whether it evokes fear and worry (i.e., dread)^24^. As noted, most respondents believed water risks during Uri to be severe (i.e., high threat). Yet, other research related to BWNs during extreme weather disasters—like Hurricane Katrina—found that individuals had low levels of perceived risk^44^. Further, Vedachalam et al.’s (2016) meta-analysis on compliance with BWNs found that, “awareness of BWA was moderately high, *except* in situations involving extreme weather,” (p. 136)^23^. Variations in risk perception around BWNs suggest a need to examine how dread risk and EPPM tenets function across typical BWN events *and* during extreme weather disasters. Such research should also continue to examine how different demographic variables impact efficacy beliefs and risk perceptions, inclusive to water quality perceptions.

Age, gender, and level of education were *not* found to influence the likelihood of whether respondents complied with BWNs during Uri, although income level was influential in increasing BWN compliance. Lai et al. reported similar findings related to income, but contradictory findings related to age and level of education, noting that “respondents who were older and had higher levels of education and income were likely to have a wider range of disaster information repertoires,” (p. 747) like emergency supplies^45^. These results highlight the need for future research as well as the need to better understand at-risk populations so that risk messages more effectively promote efficacy and acknowledge various risk perceptions^46^.

This study also suggests that current conceptualizations of risk perception may be too simplistic. Risk perception is currently conceptualized and measured primarily as a combination of severity + susceptibility for generic, nonspecific threats^18^. However, “threat” can be perceived in more complex ways than just “severity” and “susceptibility.” In the context of Uri, for example, respondents answered questions about their perceived risks surrounding water borne diseases (which are the ‘threat’ that BWNs aim to mitigate). Though this potential risk is communicated in a BWN, these questions do not link this risk specifically to the context of the ongoing disaster (i.e., Uri). Therefore, an individual’s *overall* perception of risk may be captured in the current conceptualization, but *risk in relation* to a specific risk event may not. Different conceptualization(s) may impact results, which is especially important because many disasters have cascading effects and numerous risks that emerge due to the *initial* disaster event.

During Uri, impacted populations experienced extreme winter weather, power outages, loss of heat, frozen pipes, damage to buildings and infrastructure, travel restrictions, water issues and over 200 individuals died^5^. Little research specifically involving communication has examined these forms of cascading risks^7^. When asked about risk perception surrounding water borne disease (i.e., the risk associated with BWNs), respondents may have been thinking about this risk in relation to other risks posed by Uri.

Results from this study suggest that extreme disasters can impact efficacy levels. While affected individuals may have high efficacy (self + response) outside of disaster contexts, a lack of resources due to a disaster can impact efficacy *during* the event when individuals are trying to enact protective actions^13^. As Witte purports in EPPM, individuals may have the intention to comply with a protective action—which is guided in part by perceived efficacy—but what stops them from executing the action may relate to their (lack of) skills and/or environmental constraints^13^. This nuance was exemplified during Uri, as people may have intended to boil water, but could not do so due to loss of electricity. In addition, this disaster also complicated how risk perception and efficacy function as related to messaging. Stated another way, efficacy messages that cannot be followed due to environmental factors/constraints may impact risk perception and response behaviors.

The complicated nature of sending crisis messages during Uri also influences how risk perception and efficacy typically function to influence at-risk individuals’ response behaviors. For instance, EPPM suggests that messages that individuals perceive as threatening can produce adaptive, desired responses (e.g., boiling water) when both perceived threat and efficacy are high^13,47^. Yet, when individuals cannot receive potentially “threatening messages” that signal risk (i.e., BWN), EPPM’s assumption may not hold.

Efficacious messages are not only important to help disaster-affected individuals comply with protective actions, but they are also important because they can help publics’ practice preparedness during pre-crisis times^38,48^. Further, efficacious messages can help at-risk individuals reduce uncertainty and better understand risks^49^. In these situations, efficacious messages may prompt information seeking, and increase knowledge sufficiency about the event^20^. However, the very nature of Uri complicated this action for individuals since many did not have power due to the storm (i.e., cascading risks). Information seeking, as related to efficacy, is important because it can mediate the effects of perceived susceptibility (one aspect of risk perception) and anxiety, decrease message rejection, partially mediate effects linked to perceived susceptibility and fear, and thus, potentially lead to an increase in overall message acceptance^20^. As such, future research should examine risk communication during events like Winter Storm Uri and query how affected publics’ efficacy levels are impacted by such communication.

## Methods

A non-representative, cross-sectional, survey was administered using Qualtrics XM (Qualtrics, Provo, UT). All methods were carried out in accordance with the methods approved by the Institutional Review Board (IRB) at the University of Texas at Tyler’s (IRB-FY2021-129) and Wayne State University (IRB-21-02-3278). Using the IRB-approved recruitment script, all respondents were presented an information sheet prior to their participation in this research and consented before starting the online survey. To participate in the study, respondents needed to live in Texas or Oklahoma during February 14–February 26, 2021 (Winter Storm Uri and related BWN parameters) and be at least 18 years of age. Respondents were asked to verify this information at the beginning of the survey and enter their city, state, and five-digit zip code to confirm their residence.

**Figure 1:**
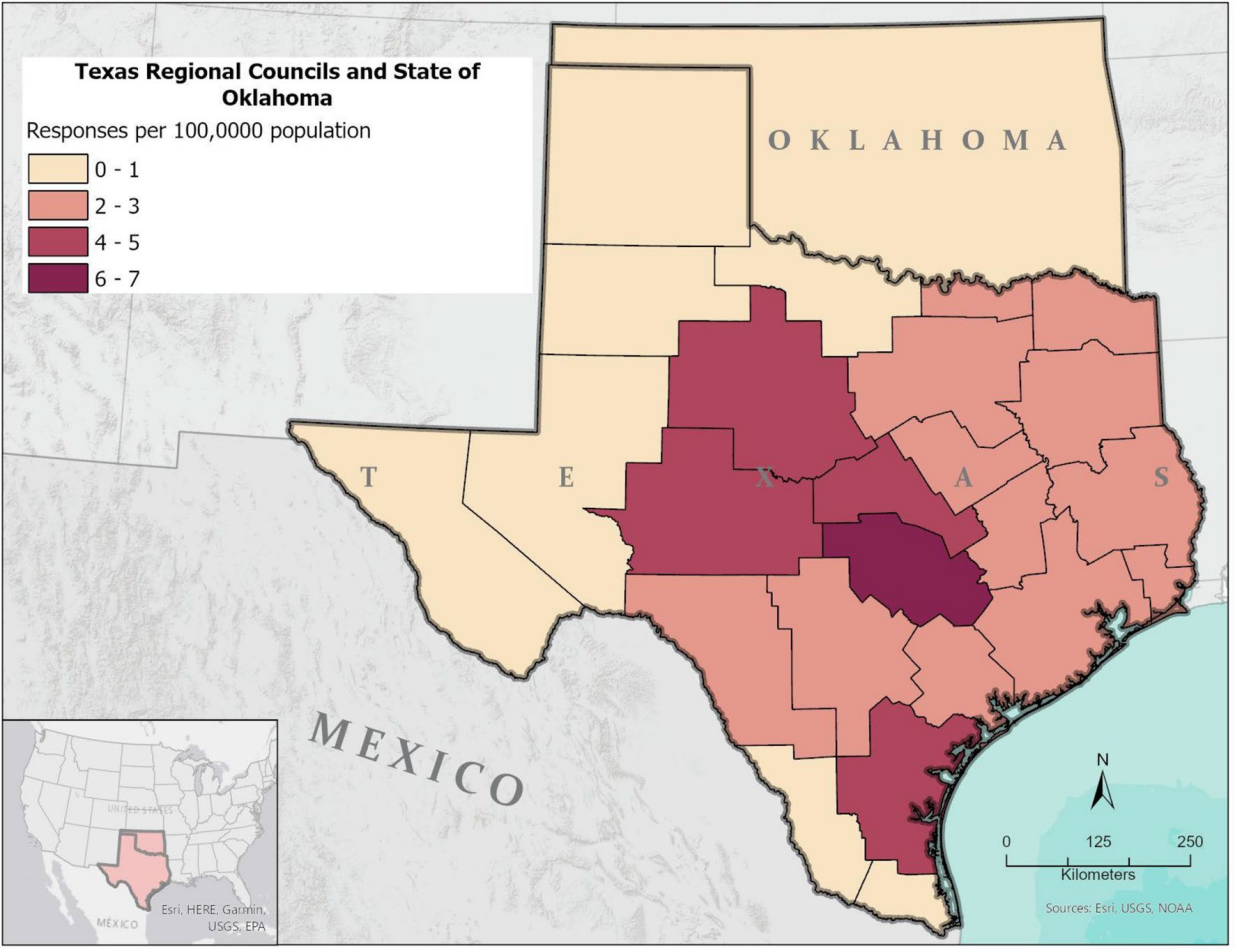
Proportion of population that reported if they did or did not boil water during winter storm Uri. Polygons inside Texas represent the 24 administrative boundaries of the Regional Councils of Governments as defined by the Texas Association of Regional Councils.

Responses from adults (18 + years old) living in Texas and Oklahoma during the Winter Storm Uri were collected March 2 through April 21, 2021 (Fig. 1). The survey took respondents approximately seven minutes (median response time) to complete. Some 99.9% of respondents reported they were affected by Uri from February 14–26, 2021. Overall, there were a total of 893 respondents; 775 from Texas, 101 from Oklahoma (including Native American reservations), and 17 other respondents^7^ (see Table 4).

**Table 4:** Demographics of respondents that indicated if they did or did not boil their water^7^.

Respondent characteristics	Number of respondents (% sample)	Fraction of Texas and Oklahoma residents (%)
Gender (N = 650)		
Male	63 (9.7)	49.6^a^
Female	573 (88.2)	50.4^a^
Trans, Genderqueer, and Other	14 (2.2)	NA
Age (N = 648)		
18–24 years	39 (6.0)	9.7^a^
25–44 years	257 (39.7)	28.1^a^
45–64 years	271 (41.8)	23.6^a^
65–74 years	69 (10.6)	8^a^
75 Years or over	12 (1.9)	5.3^a^
Education (N = 648)		
Less than High School	1 (0.2)	14.9^a^
High School Diploma	20 (3.1)	26.0^a^
Some College	149 (23.0)	28.9^a^
Bachelor’s Degree or Higher	478 (73.8)	30.2^a^
Annual Family Income (N = 570)		
Less than $34,999	88 (15.4)	27.4^a^
$35,000-$49,999	67 (11.8)	12.5^a^
$50,000-$74,999	123 (21.6)	18.0^a^
$75,000-$99,999	107 (18.8)	12.6^a^
$100,000 or more	185 (32.5)	29.5^a^
Hispanic or Latino	NR*	36^b^
Non-Hispanic or Latino Race (N = 593)		64^b^
White	554 (93.4)	66^b^
Black	12 (2.0)	17.6^b^
Asian	0 (0)	7.8^b^
Mixed race	14 (2.4)	5.9^b^
American Indian	13 (2.2)	1.9^b^

*N* Sample size, *NR* Not recorded.

*Due to a survey error, the number of respondents that identified as Hispanic or Latino was not recorded.

^a^US Census (2019) American Community Survey 1-Year Estimates Selected Population Profiles (Dataset: ACSSPP1Y2019).

^b^US Census (2020) Decennial Census Redistricting Data (PL 94-171) (Dataset: DECENNIALPL2020).

Data collection began via snowball sampling and a targeted Facebook advertisement campaign and occurred from March to May 2021. For snowball sampling, researchers posted the survey link on their social media pages. Using the IRB-approved recruitment script, researchers also asked their social networks to take and/or share the survey. Additionally, a paid-for-advertisement campaign was placed on Facebook to promote the survey to individuals in Texas and Oklahoma based on user data. The advertisement promoted the IRB-approved recruitment script and the Qualtrics link. Researchers also shared the survey link with their non-social media social networks, such as university colleagues and academic communities.

Risk perception was calculated as the product of susceptibility and severity, using four adapted items from Rimal and Real^18^. Following Rimal and Real^18^, response scores for four questions focused on perceived susceptibility to and severity of waterborne disease were averaged to provide an indexed measure of *risk perception.* Additionally, the average scores for *susceptibility* and *severity* were also investigated individually to determine how these perceptions influence risk-mitigating behavior. Perceptions of water quality were assessed with three questions adapted from the AWWA survey on public perceptions of tap water safety^37^. Because the scales for responses to these three questions were different, the scores were normalized and averaged to constitute a *water quality perception* measure. Six items from Witte et al.’s^36^ Risk Behavior Diagnosis Scale were used to assess perceived efficacy related to risk. Average scores for the six questions were calculated and used as a continuous variable defining *perceived efficacy*. Additional details regarding survey questions are presented in the SI. Respondents were asked to indicate gender identity, age, race, number of people in their household, if children live in the household, family income, level of education, and employment status^50,51^.

### Statistical analysis

All statistical analyses were performed in SPSS (Version 29, IBM). Hypothesis 1 (“Previous experience with a BWN(s) will alter individuals' perceptions of water quality and the risk of acquiring a waterborne disease during Uri”) was evaluated using Mann–Whitney U tests, because these data are not normally distributed. Hypothesis 2 (“Higher levels of perceived efficacy will be related to individuals’ perceptions of water quality and the risk of acquiring a waterborne disease during Uri”) was evaluated by assessing correlations between perceived efficacy and perceptions of risk and water quality. Because variables investigated included those that are not normally distribution (e.g., water quality perception) and ordinal (e.g., family income) the non-parametric Kendell’s tau b (${\tau }_{B}$) was used to assess correlations relevant to H2.

Hypothesis 3 (“Perceptions of efficacy, water quality, and risk will influence compliance with BWNs during Uri”) was evaluated using a series of binary logistic regression models. Only respondents that received a BWN or similar notification were included in this analysis. The likelihood that respondents boiled water was based on the general equation:

$$logit={L}_{i}=ln\left(\frac{{P}_{1}}{1-{P}_{1}}\right)={\beta }_{0}+{\beta }_{1}{x}_{1,i}+\dots +{\beta }_{k}{x}_{k,i}$$

where ${L}_{i}$ is the odds that a survey respondent boils water; P_1_ is the probability of outcome 1 (i.e. the respondent boils water); ${x}_{k, i}$ are predictor variables such as perceptions of risk, water quality, and efficacy, and respondents’ gender, education, age, income, ability to boil water, living with minor(s), and previous BWNs experience; ${\beta }_{k}$ are fitted coefficients; and *k* is the number of predictors. For regression models, perceptions of risk, water quality and efficacy are treated as continuous variables. More information regarding collinearity of model predictors can be found in the SI.

### Limitations

Several limitations should be considered when interpreting these results. First, respondents were recruited through Facebook advertisements. While this limited potential respondents to those who utilize and have access to Facebook, it allowed for sampling of the disaster-affected population (Texas and Oklahoma) through ads targeting users’ geo-location tool. Further, this method of recruiting provided an expedient way of reaching respondents soon after Uri while many were still under a BWN. Second, to protect respondent privacy, rather than asking respondents for their street level address, the survey only requested respondents identify their city, state and zip code. Therefore, our analysis was limited to matching responses to the smallest spatial unit possible, which typically was the city. This limitation prevented us from performing further spatial analyses or incorporating other census information that might have enhanced our understanding of local conditions that may have influenced respondent responses to BWNs. Third, while widely used and well-established, the risk perception survey items had low reliability scores in this study. Though the survey items asked respondents about future risk, the low reliability may be related to individuals responding based on current feelings of susceptibility, due to being under a BWN at the time. It is also possible that some respondents may have considered frozen pipes, a lack of resources and power, BWNs, and other cascading effects from Uri as a singular risks event rather than viewing them as individual risks emerging from a larger disaster. Thus, measuring risk perception *during* an ongoing disaster may require a more nuanced approached. Future research should further examine such an approach.

### Supplementary Information

Supplementary Information.

## Data Availability

Data evaluated during this study is archived at Open Data at Wayne State and can be found at https://doi.org/10.22237/waynestaterepo/data/1685726763.
