# Supplementary material for: Boil water notices as health-risk communication: risk perceptions, efficacy, and compliance during winter storm Uri
Source: Sci Rep. 2024 Jan 8;14:850. doi: 10.1038/s41598-023-50286-y (PMC10774435; doi:10.1038/s41598-023-50286-y)
Supplement: Supplementary file 1 — Supplementary Information. [file 41598_2023_50286_MOESM1_ESM.docx]

**Boil Water Notices as Health-Risk Communication: Risk Perceptions, Efficacy, and Compliance during Winter Storm Uri**

**Supplemental Information**

Ashleigh M. Day, PhD*

Assistant Professor, School of Communication, Northern Arizona University
Email: Ashleigh.Day@nau.edu
ORCID: 0000-0003-3212-2611

Sydney O'Shay, PhD
Assistant Professor, Department Communication Studies and Philosophy, Utah State University
Email: sydney.oshay@usu.edu
ORCID: 0000-0002-6880-4561

Khairul Islam, PhD

Assistant Professor, Department of Communication Studies, State University of New York at Oswego

Email: khairul.islam@oswego.edu

ORCID: 0000-0001-7624-0041

Matthew W. Seeger, PhD
Professor, Department of Communication, Wayne State University
Email: matthew.seeger@wayne.edu
ORCID: 0000-0002-5585-3081

F. Gianluca Sperone, MA, MS
Assistant Professor, Department of Environmental Science & Geology, Wayne State University

Doctoral Candidate, Department of Civil and Environmental Engineering, Wayne State University

Email: fgsperone@wayne.edu

ORCID: 0000-0002-6394-3984

Shawn P. McElmurry, PhD, PE*
Professor, Department of Civil and Environmental Engineering, Wayne State University
Email: s.mcelmurry@wayne.edu
ORCID: 0000-0001-7398-431X

Funding:

This work was conducted as part of the Water and Health Infrastructure Resilience and Learning (WHIRL) project funded by the National Science Foundation (NSF) under Grant Numbers CBET-1832692. The survey was performed in accordance with University of Texas at Tyler’s Institutional Review Board polices (#IRB-FY2021-129). The content is solely the responsibility of the authors and does not necessarily represent the official views of the NSF or other WHIRL collaborators.

**Supplemental Information**

In this Supplementation Information (SI) document, additional information is presented about extant research on risk and crisis communication, risk perception, and boil water advisories. Methods and survey design are also further described in this SI.

**Risk Communication**

Risk communication has been defined as “the systematic dissemination of information to diverse audiences (e.g., individuals, communities, and institutions) facilitating their informed, independent decision making about the existence, nature, and/or severity of risks and hazards affecting health, safety, and the environment,” [36]. Risk communication is most often associated with the pre-crisis context, while a similar process used *during* an emergency to help mitigate harm is often described as crisis communication [37]. Risk communication should be interactive, science-based, and culturally appropriate and risk communicators should tailor messages to their audiences, their preferred informational sources, and their preferred media uses [39, 40]. Boil water notices (BWNs) should function as risk message. BWNs, for example, are often issued during pre-crisis times in response to low pressure in water distribution systems, but are also issued during disasters, such as floods, that contaminate treatment plants. A variety of factors are associated with the effectiveness of the informative *and* persuasive features of risk communication [37, 38]. One important factor that must be considered in risk perception.

As noted in the primary manuscript, risk perception is the general feeling of being vulnerable to a *possible* threat [14]. While risk perception can be conceptualized many ways, Rimal and Real [14] conceptualize risk perception as a combination of a person’s perceived susceptibility and perceived severity of a risk and these constructs are assessed based on threats that may occur in the *future*. Understanding risk perception is essential to crafting tailored and effective risk communication. However, risk perception can be greatly influenced by demographic variables and, thus, necessitates that risk communicators understand the interactive dynamic among risk perception, demographics, and how these impact risk communication exigencies and subsequent decision-making among at-risk populations.

Differences among demographic variables have been shown to impact individuals’ communicative needs, decision-making, and actions during risk and crisis events. For instance, during the Flint, Michigan water crisis, women were more likely than men to use social media (e.g., Facebook, Instagram) to seek information about the crisis [51]. Moreover, African American respondents were more likely than other racial groups to prefer to receive information about the crisis from interpersonal networks and on Instagram [52]. In other contexts, such as natural disasters like the 2017 Hurricane Harvey, identity as a “pet owner” influenced individuals’ decision making during a disaster, even if staying with their pet compromised their own safety [71, 72]. Understanding how demographic characteristics impact communicative needs, actions, and decision-making during risk and crisis events is imperative for risk communication, especially for water-related risks.

***Water and Risk Communication***

To date, there is limited risk communication research on water issues. However, water risks are common and are only projected to increases as the global population increases [41, 42]. Common water risks that are experienced globally include: limited access to clean water, too much water, contamination, disruptions to freshwater systems, sanitation, and overall water security and sustainability [41]. When water is contaminated, polluted, and/or lacks appropriate treatment, risk communication becomes an integral part of mitigation and, ultimately, helps protect public health and safety. A specific form of risk communication, in these instances, is a BWN.

***Boil Water Notices***

BWNs are risk messages [7] issued when there are reasons to believe that water quality is or may be compromised—including low pressure from broken pipes or severe weather that impacts the power grid, like Winter Storm Uri. BWNs are warnings to inform and persuade the public to boil their municipal-supplied water before consumption or use [10]. When communicated effectively, BWNs may help mitigate potential harm and help individuals who are at-risk make informed decisions and successfully enact protective actions [8, 9].

Despite awareness of an active BWN, compliance with suggested protective actions varies among affected populations from 36% to 98% [8]. When affected populations learn of a BWN from multiple sources, such as the internet plus interpersonal networks, they are more likely to follow protective actions [12]. People may also be more likely to comply with protective actions when at least one other person in the household is also taking these actions [12]. Common reasons for non-compliance during a BWN include forgetfulness, perceived inconvenience, appearance of clean water, and not believing the initial notification of a BWN [11, 12, 43]. Overall, low risk perception is consistently reported as a primary reason for non-compliance [11, 12].

Communication of BWNs becomes more complicated when a winter storm, such as Uri, unravels the power grid impacting communication systems. Moreover, lack of power may impede the ability of people to boil their water [4, 6, 44]. Since much of Texas was impacted by power outages and approximately 40% of Texans were under a BWN following Uri, it is likely that many did not receive the BWNs in a timely manner or perhaps at all [6, 45, 46]. With most communication technologies impacted by the lack of power, communicating BWNs to individuals at-risk was challenging.

***Efficacy***

Both self- and response-efficacy are important to compliance with recommended protective actions. Additionally, both self- and response-efficacy typically increase when an individual seeks credible information about a risk or crisis [32]. However, during an extreme weather event—like Uri—that dismantles a state’s power grid, information seeking is hindered as well as the ability to disseminate risk messages.

According to the Extended Parallel Process Model (EPPM), “efficacy” is a combination of self- and response-efficacy [17, 33]. *Response efficacy* (i.e., outcome expectations) accounts for an individual’s belief that enacting a specific (protective) action will result in the changes they seek, such as boiling water will produce safe, clean water for consumption [47]. Perceived *self-efficacy*, more specifically, is one’s belief that they have the capabilities to enact the suggested action, such as being able to boil water on their stovetop [48]. “Capabilities” within self-efficacy may also include an individual’s access to resources necessary to enact a recommended protective action, such as having pots to boil water or having electricity for their stovetop to boil water. This knowledge is important because individuals with a high level of perceived self-efficacy are often able to translate knowledge into behavior [48]. While individual efficacy measures are important to consider, efficacy promoted via risk communication is also an important consideration.

Based on EPPM propositions, the effectiveness of risk communication is highly contingent on whether efficacy information is effectively conveyed [17]. When risk communication fails to convey efficacy to target populations, they are likely to focus on controlling their fear versus controlling for the risk [17]. However, the low rate of efficacious messages during risk and crisis events is concerning. For instance, during the 2014-2015 Ebola outbreak, risk messages in radio programming as well as newspaper content included relatively low levels of both self- and response-efficacy [49]. The low levels of efficacious messages limit the ability of the public to take protective action. Furthermore, media tend to effectively communicate *risk* to the public, but are much less effective in communicating *efficacy* [50]. While mass media have a significant role in disseminating risk communication, other communicators also play a role, such as public officials distributing messages through interpersonal networks, etc. [51, 52].

***Efficacy and Risk Perception***

Efficacy plays a role in an individuals’ perceived risk. Risk perception is “the belief that one is vulnerable” to some risk such as disease, danger, etc. [14]. Whereas risk perception alone is not related to behavioral intentions, efficacy interacts with risk perception to help predict health behaviors [53]. Research suggests that among individuals with some level of perceived risk, those who have higher levels of efficacy experience more positive health outcomes than individuals with lower levels of efficacy [14]. Further, high risk perception and high efficacy beliefs are positively related to information-seeking intentions and behaviors [49]. Individuals with lower efficacy beliefs *may* seek information about a risk but are less likely to retain that information due to anxiety [49].

The presence of children in the home impacts individuals’ risk perceptions and behaviors related to potential health threats. Families with children perceive high risk around threats across contexts such as COVID-19 [73], environmental health risks [74], and perfluoroalkyl substances (PFAS) in water [75]. Individuals with children that have special healthcare needs and families with more than two children perceive higher risk around environmental health threats than their counterparts [76]. Families with children also seek information related to potential threats at higher rates during crises (than families without children) [51]. Further, caregiver knowledge and efficacy impact preparedness for potential disasters [77]. For example, Latina mothers described fear, a lack of knowledge around risks, and a lack of support in their home and community as barriers to taking protective actions despite a desire to protect their families from potential risks [74]. Thus, individuals with children in the home perceive risk and experience and respond to health threats in unique ways when compared with individuals/families without children.

Americans have consistently expressed concerns about contamination of drinking water with 83% reporting a “great deal” or “fair” amount of worry in 2022 [13]. Perceptions about the quality and safety of water may interact with risk perceptions and efficacy beliefs to impact decisions to take protective actions. For example, among people who did *not* comply to a recent BWN in Norway, the principal reason for non-compliance was a perception of low or no risk of getting sick from the water [11]. Similarly, private well users in Ireland reported low risk perception and low self-efficacy related to lack of knowledge and accessibility to services as well as the high costs of testing well-water despite recent flooding events that could cause contamination [54]. Further, private-well users who had high risk perceptions regarding water contamination and perceived that they had some control over contamination risks were more likely to properly maintain their private-well [55]. Lastly, a lack of understanding around potential water contamination and recommended actions led residents impacted by the 2018 Camp Fire in California to continue using their tap water despite restrictions and/or to behave in ways that were inconsistent with public health recommendations [56]. For instance, of those who continued to use their tap water, 75% used an in-home filtration system although the county health department recommended against them [56]. Yet, other studies have reported that previous experience with a particular risk or hazard may increase risk perception and, thus, prompt individuals to prepare for future events. Lazo et al. noted that, “Past experience with a hazard is generally thought to influence one’s recognition that a risk exists and increases motivation to protect one-self,” [15]. Simply, previous experience with a risk may increase risk perception enough to encourage positive protective actions. Overall, risk perception and efficacy beliefs play a role in people’s decisions to take protective actions (or not) when experiencing potential water contamination.

**Methods**

The survey was composed of various questions to assess respondents’ experience with BWNs, what protective actions they took in response to these events, and their risk and water quality perceptions related to BWNs. In total, there were 45 questions, and some questions include skip-logic sub-questions depending on the respondents’ answer to the earlier question(s).

Questions asked respondents about their source of drinking water and if their household was under a BWN or a “do not drink” order following Uri (approximately between February 14 to February 26, 2021). Respondents were asked if they had running water during the advisory and the specific ways in which water in their home was impacted by the winter storm (e.g., low pressure, discolored water). All responses in this section were categorical (i.e., non-scale items).

To collect contemporaneous Uri data in a timely manner, researchers modified extant survey instruments for this research. The employed survey instruments have been utilized in other crisis events and, thus, have demonstrated validity and reliability (see further details under “survey”).

Risk perception was calculated as the product of susceptibility and severity, using four adapted items from [14]. There were two questions about perceived susceptibility: “Compared to most people my age, I understand that my risk of getting a water borne disease is…” and “The likelihood of my getting a water borne disease is…” These two questions used a scale ranging from (1) extremely low to (5) extremely high. To assess perceived severity, respondents were asked the following two questions: “Water borne diseases are serious diseases that can cause harm,” and “Water borne diseases are more serious than most people realize,” using a scale ranging from (1) very strongly disagree to (5) very strongly agree. (The structure of these questions and bar graphs are noted below).


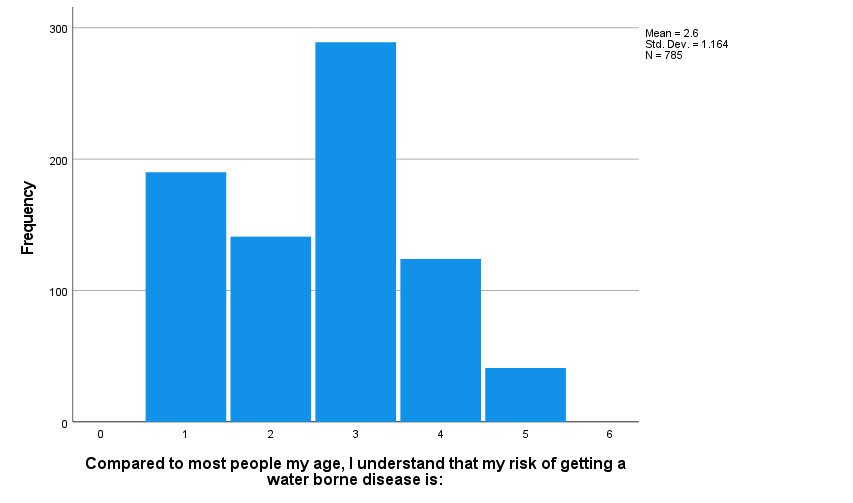
Q38_1SUSCEPTIBILITY1 - Compared to most people my age, I understand that my risk of getting a water borne disease is:

1. Extremely Low
2. Below Average
3. Neutral
4. Above Average
5. Extremely High


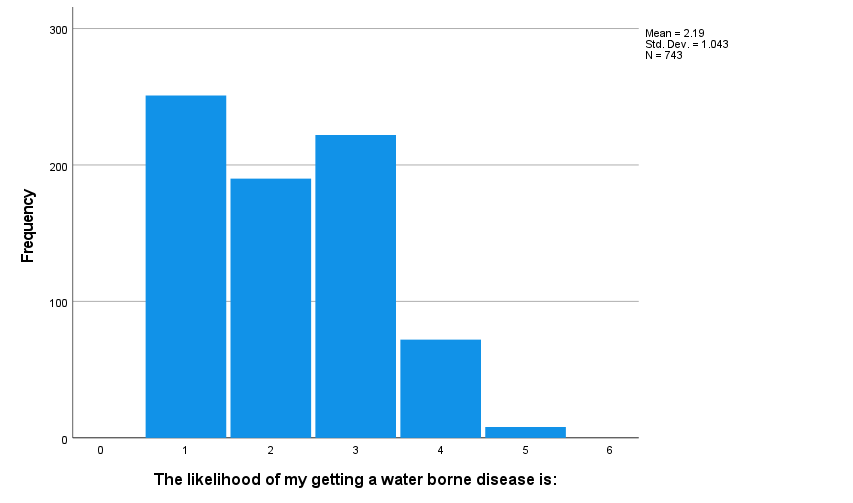
Q38_2SUSCEPTIBILITY2 - The likelihood of my getting a water borne disease is

1. Extremely Low
2. Below Average
3. Neutral
4. Above Average
5. Extremely High

Q39_1SEVERITY1 - Water borne diseases are serious diseases that can cause harm.

1.
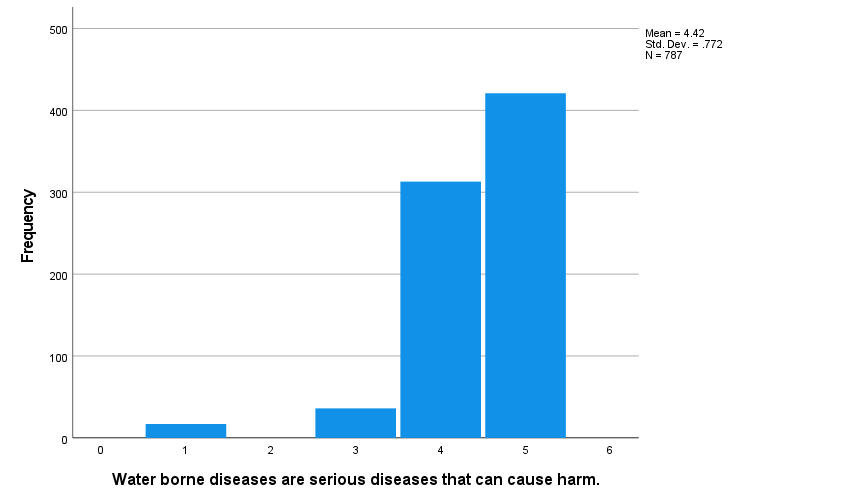
Very Strongly Disagree
2. Disagree
3. Neither Agree or Disagree
4. Agree
5. Very Strongly Agree

Q39_2SEVERITY2 - Water borne diseases are more serious than most people realize

1.
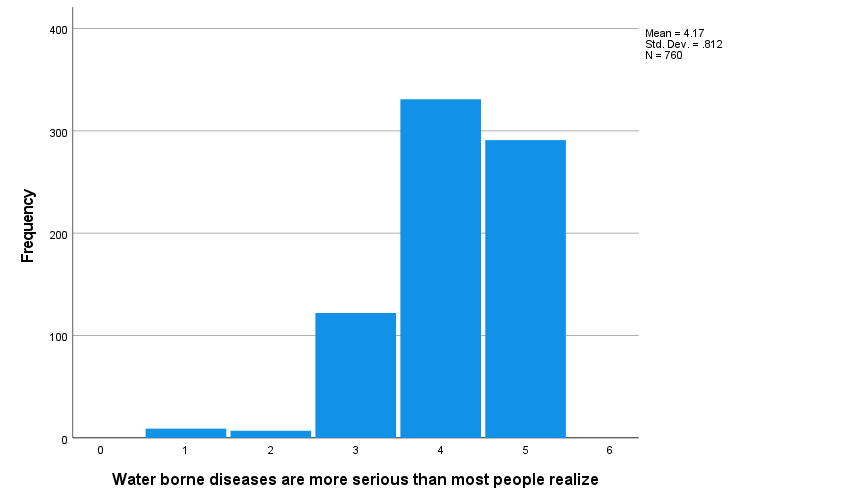
Very Strongly Disagree
2. Disagree
3. Neither Agree or Disagree
4. Agree
5. Very Strongly Agree

Although these items are well established in the literature, in the context of our study, these four items exhibited low reliability (Cronbach’s α = 0.57). However, it is not uncommon that researchers to group questions with Cronbach’s α = 0.57 [60] although this can inhibit accurate model predictions. Following [14], response scores for these four questions were averaged to provide an indexed measure of risk perception. Additionally, the average scores for susceptibility and severity were also investigated individually to determine how these perceptions influence risk mitigating behavior.

The second way we attempted to define risk perception was based on the following three questions, adapted from the American Water Works Association survey [16]. The three questions focused on respondents’ perception of water quality at the faucet, their satisfaction with faucet water, and their perception of the safety of the water from their faucet were used for this second measure.


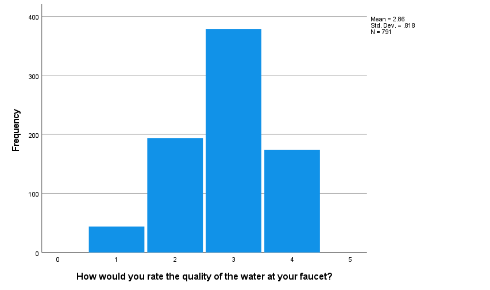
Q35BWAPERCEPTIONS1 - How would you rate the quality of the water at your faucet?

1. Poor
2. Just fair
3. Good
4. Excellent


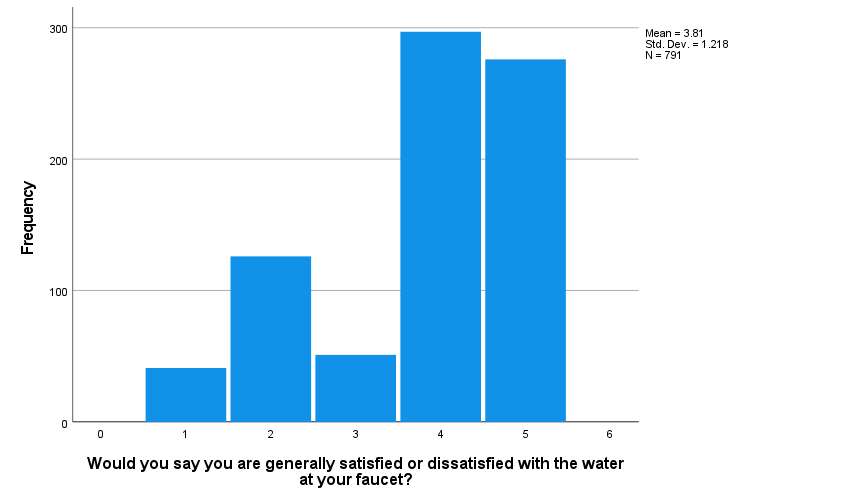
Q36BWAPERCEPTIONS2 - Would you say you are generally satisfied or dissatisfied with the water at your faucet?

1. Very unsafe
2. Somewhat unsafe
3. Do not know/unsure
4. Somewhat safe
5. Very safe

Q37rBWAPERCEPTIONS3 - In your view, how safe or unsafe is the water at your faucet?

1.
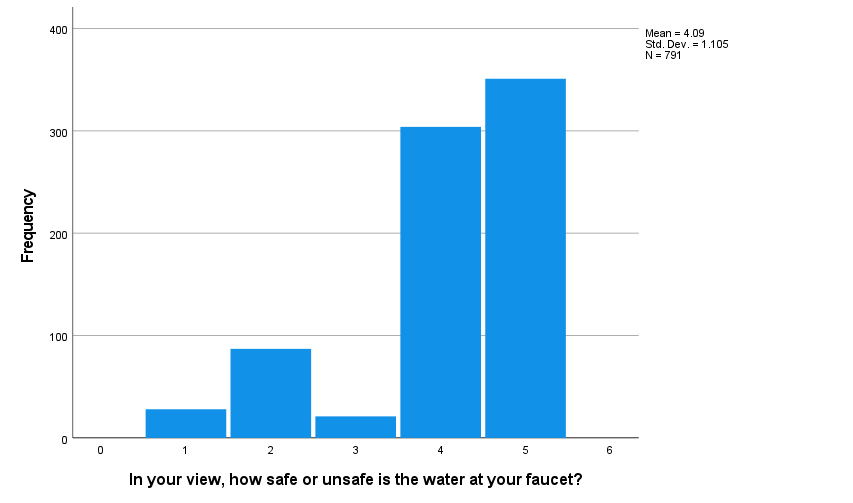
Very unsafe
2. Somewhat unsafe
3. Do not know/unsure
4. Somewhat safe
5. Very safe

Because the scale for the first question (Q35BWAPERCEPTIONS1) used a 4-point scale, this was normalized to a 5-point scale and then the average of the 3 scores was computed to constitute Risk Perception Measure 2. Reliability for these items was higher (Cronbach’s α = 0.83) than risk perception.

In this conceptualization, perceived efficacy is comprised of response efficacy *and* self-efficacy [33]. For response efficacy, respondents were asked to respond to the following three questions: “Water advisories work in preventing water borne diseases,” “Following a water advisory is effective in preventing water borne diseases,” and “If I follow a water advisory, I am less likely to get a water borne disease.” For self-efficacy, respondents were asked the following three questions: “I am able to follow a water advisory to prevent getting a water borne disease,” “Following a water advisory is easy to prevent getting a water borne disease,” and “Following a water advisory to prevent water borne diseases is convenient.” This set of six questions used a five-point scale, ranging from (1) strongly disagree to (5) strongly agree (*Cronbach’s α* = 0.71).

**Data Analysis**

We took two different approaches to assess reliability of the scaled variables (i.e., risk perception and efficacy). Initially, the calculation of Cronbach’s alpha coefficients was conducted to assess internal consistency. The alpha reliability was deemed sufficient, except for risk perception (α = 0.57). Because of the low scale reliability, we also measured the two components that constituted risk perception individually as well as other items that were likely to influence behavior. This approach allowed us to compare the findings from two separate analyses.

***Binary Logistic Regression Model***

Ideally, we would have no relationship between predictor variables included in logistic regression. Models 1-3 (Table 3) focus on assessing the perception of water quality and risk on whether respondents boil their water. In this case, the most important interaction that is of concern is between predictors that are related to *water quality* or *risk*. The largest correlation observed between predictor variables is between *risk perception* and *perceived water quality* (r^2^= 0.067; note this is not the square of Kendall’s tau b, which is nonparametric and cannot be squared to obtain the coefficient of determination, r^2^). This correlation translates to a variable inflation factor (VIF) of 1.073, meaning the regression coefficient (β) may be inflated by 7.3% based on this relationship. While this amount of inflation was considered acceptable, we still offer a Model 3 which separates these two variables for just this reason. The next highest correlation is between family *income* and *perceived water quality* (r^2^= 0.032) has a VIF of 1.033. A 3.3% error in the estimated β for *perceived water quality* is very small and deemed acceptable error. Additionally, an elevated VIF for an ordinal variable with a small reference level is to be anticipated. In this case we have 5 levels of *family income* and only 15.3% of respondents indicated they were in the lowest reference level. Compared to the highest income level (>$100k) which was associated with 33.7% of respondents, an elevated VIF is expected. Finally, the large sample size (n>700) also decreases the likelihood that the low levels of collinearity observed have a significant impact on regression estimates.

The logistic regression models (Models 4-6) presented in Table 4 have similar low VIFs. The largest VIF observed is between the two variables that are most of interest, *susceptibility* and *perceived water quality* variables (VIF=1.122). Because of this interaction, Models 6 was developed to avoid this interaction. Importantly, the regression coefficient ($\beta\approx$-0.2) was virtually unchanged for the *susceptibility* variable between these two models. The next highest VIF was 1.043 between *family income* and *susceptibility*. For the same reasons described above, a possible 4.3% inflation on the regression coefficient was deemed acceptable.

**REFERENCES**

1. Weather.com. (2021, February 16). Winter storm Uri spread snow, damaging ice from cost-to-coast, including the Deep South (recap). Retrieved from <https://weather.com/safety/winter/news/2021-02-14-winter-storm-uri-south-midwest-northeast-snow-ice>
2. Houston Advanced Research Center [HARC]. (2021). Winter Storm Uri’s impact & pathways to resilience in Texas. Retrieved from <https://experience.arcgis.com/experience/cc48fcfebfae414b99b3d18f86c72c27>
3. Houston Advanced Research Center [HARC]. (2021). Winter Storm Uri’s impact & pathways to resilience in Texas: Water supply impacts. Retrieved from <https://experience.arcgis.com/experience/cc48fcfebfae414b99b3d18f86c72c27/page/page_36/?views=view_8>
4. Texas Commission of Environmental Quality [TCEQ]. (2021). Severe winter weather event 2021 - Public water system boil water notice list: public water systems under boil water notice (BWN). Retrieved February 19, 2021 from <https://www.tceq.texas.gov/downloads/response/temporary-suspension-of-rules-due-to-severe-weather/boil-water-notice-list.pdf>
5. Glazer, Y. R., Tremaine, D. M., Banner, J. L., Cook, M., Mace, R. E., Nielsen-Gammon, J., ... & Webber, M. E. (2021). Winter Storm Uri: A Test of Texas’ Water Infrastructure and Water Resource Resilience to Extreme Winter Weather Events. *Journal of Extreme Events*, 2150022.
6. Texas Commission of Environmental Quality [TCEQ]. (2021). After-action review of public water systems and Winter Storm Uri. Retrieved from <https://www.tceq.texas.gov/drinkingwater/after-action-review>
7. Day, A. M., Islam, K. O’Shay, S., Taylor, K., McElmurry, S. P., & Seeger, M. W. (2022). Consumer response to boil water notifications during Winter Storm Uri. *Journal AWWA*, *114*(5), 26-33. <https://doi.org/10.1002/awwa.1919>
8. Harding, A. K., & Anadu, E. C. (2000). Consumer response to public notification. *Journal-American Water Works Association*, *92*(8), 32–41. https://doi.org/10.1002/j.1551-8833.2000.tb08989.x
9. Sorensen, J. H. (2000). Hazard warning systems: Review of 20 years of progress. *Natural Hazards Review*, *1*(2), 119–125. https://doi.org/10. 1061/(ASCE)1527-6988
10. Vedachalam, S., Spotte-Smith, K. T., & Riha, S. J. (2016). A meta-analysis of public compliance to boil water advisories. *Water Research*, *94*, 136–145. <https://doi.org/10.1016/j.watres.2016.02.014>
11. Franer, K., Meijerink, H., & Hyllestad, S. (2020). Compliance with a boil water advisory after the contamination of a municipal drinking water supply system in Norway. *Journal of Water and Health, 18*(6), 1084-1090. https://doi.org/10.2166/wh.2020.152
12. Karagiannis, I., Schimmer, B., & de Roda Husman, A. M. (2009). Compliance with boil water advice following a water contamination incident in the Netherlands in 2007. *Eurosurveillance, 14*(12), 1-3. http://www. eurosurveillance.org/ViewArticle.aspx?ArticleId=19156
13. Gallup (2023). Environment. Retrieved from: https://news.gallup.com/poll/1615/environment.aspx
14. Rimal, R., & Real, K. (2003). Perceived risk and efficacy beliefs as motivators of change: Use of the risk perception attitude (RPA) framework to understand health behaviours. *Human Communication Research*, 29(3), 370–399.
15. Lazo, J. K., Bostrom, A., Morss, R. E., Demuth, J. L., & Lazrus, H. (2015). Factors affecting hurricane evacuation intentions. *Risk Analysis, 35*(10), 1837-1857.
16. American Water Works Association. (2020). AWWA public perceptions of tap water. Retrieved February 18, 2021, from https://www.awwa.org/Portals/0/AWWA/Communications/23001PDFEdits-1.pdf
17. Witte, K. (1994). Fear control and danger control: A test of the extended parallel process model (EPPM). *Communication Monographs, 61,* 113–134. <https://doi.org/10.1080/03637759409376328>.
18. Barnett, D., Thompson, C., Semon, N., Errett, N., Harrison, K., Anderson, M., . . . Storey, J. (2014). EPPM and willingness to respond: The role of risk and efficacy communication in strengthening public health emergency response systems. *Health Communication,* *29*(6), 598-609.
19. Smith, S., Rosenman, K., Kotowski, M., Glazer, E., McFeters, C., Keesecker, N., & Law, A. (2008). Using the EPPM to Create and Evaluate the Effectiveness of Brochures to Increase the Use of Hearing Protection in Farmers and Landscape Workers. *Journal of Applied Communication Research,* *36*(2), 200-218.
20. Zarghami, F., Allahverdipour, H., & Jafarabadi, M. (2021). Extended parallel process model (EPPM) in evaluating lung Cancer risk perception among older smokers. *BMC Public Health,* *21*(1), 1872.
21. Birmingham, W., Hung, M., Boonyasiriwat, W., Kohlmann, W., Walters, S., Burt, R., . . . Kinney, A. (2015). Effectiveness of the extended parallel process model in promoting colorectal cancer screening. *Psycho-oncology,* *24*(10), 1265-1278.
22. Witte, K. (1992). Putting the fear back into fear appeals: The extended parallel process model. *Communication Monographs, 59*, 329–349.
23. Slovic, P., Fischhoff, B., & Lichtenstein, S. (2001). Facts and fears: Understanding perceived risk. In P. Slovic (Ed.), *The perception of risk* (pp. 220–231). Sterling, VA: Earthscan. (Original work published 2000).
24. Slovic, P. (1987). Perception of risk. *Science*, *236*, 280–285.
25. Ram, P. K., Blanton, E., Klinghoffer, D., Platek, M., Piper, J., Straif-Bourgeois, S., Bonner, M.R., Mintz, E.D. (2007). Household water disinfection in Hurricane- affected communities of Louisiana: implications for disaster preparedness for the general public. *Am. J. Public Health, 97* (S1), 131-135.
26. Lai, C. H., Chib, A., & Ling, R. (2018). Digital disparities and vulnerability: mobile phone use, information behaviour, and disaster preparedness in Southeast Asia. *Disasters*, *42*(4), 734-760.
27. Seeger, M. W. (2006). Best practices in crisis communication: An expert panel process. *Journal of applied communication research*, *34*(3), 232-244.
28. Thrasher, J. F., Swayampakala, K., Borland, R., Nagelhout, G., Yong, H. H., Hammond, D., Bansal-Travers, M., Thompson, M., & Hardin, J. (2016). Influences of self-efficacy, response efficacy, and reactance on responses to cigarette health warnings: A longitudinal study of adult smokers in Australia and Canada. *Health communication*, *31*(12), 1517–1526. <https://doi.org/10.1080/10410236.2015.1089456>
29. Turner, M., Kamlem, T., N. Rimal, R., Shaikh, H., & Ume, N. (2021). Overlooking the obvious: Communication of efficacy by them mass media during the Ebola crisis in Liberia. *Prevention Science,* *22*(2), 259-268.
30. O’Shay, S., Day, A. M., Islam, K., McElmurry, S. P., & Seeger, M. W. (2020). Boil water advisories as risk communication: Consistency between CDC guidelines and local news media articles. *Health Communication, 37*(2), 152-162*.* <https://doi.org/10.1080/10410236.2020.1827540>
31. Evensen, D. T., & Clarke, C. E. (2012). Efficacy information in media coverage of infectious disease risks: An ill predicament? *Science Communication, 34*(3), 392–418. https://doi.org/10.1177/ 1075547011421020.
32. So, J., Kuang, K., & Cho, H. (2019). Information seeking upon exposure to risk messages: Predictors, outcomes, and mediating roles of health information seeking. *Communication Research,* *46*(5), 663-687.
33. Witte, K., Cameron, K. A., McKeon, J. K., & Berkowitz, J. M. (1996). Predicting risk behaviors: Development and validation of a diagnostic scale. *Journal of Health Communication,* *1*(4), 317-342.
34. Centers for Disease Control and Prevention. (2022c, January 13). Surveys in Q-bank. Retrieved from <https://wwwn.cdc.gov/qbank/Surveys.aspx#/Surveys>
35. Gender Identity in U.S. Surveillance [GenIUSS] Group. (2014). Best practices for asking questions to identify transgender and other gender minority respondents on population-based surveys. Los Angeles, CA: The Williams Institute.
36. DiClemente, R. J., & Jackson, J. M. (2016). Risk communication. In *International Encyclopedia of Public Health* (pp. 378-382). Elsevier.
37. Reynolds, B., & Seeger, M. W. (2005). Crisis and emergency risk communication as an integrative model. *Journal of Health Communication, 10,* 43–55.
38. Balog‐Way, D., McComas, K., & Besley, J. (2020). The evolving field of risk communication. *Risk Analysis*, *40*(S1), 2240-2262.<https://doi.org/10.1111/risa.13615>
39. Covello, V. T. (2007). *Effective risk and crisis communication during water security emergencies: Summary report of EPA sponsored message map- ping workshops*. National Homeland Security Research Center, Office of Research and Development, US Environmental Protection Agency. http://purl.access.gpo.gov/GPO/LPS82792
40. Sellnow, T. L., Ulmer, R. R., Seeger, M. W., & Littlefield, R. S. (2009). *Effective risk communication: A message-centered approach*. Springer.
41. Organisation for Economic Co-operation and Development (OECD). (n.d.). Water risks, disasters and climate change. Retrieved from oecd.org/water/risks-disasters-and-climate-change.htm
42. World Health Organization (WHO). (2019, June 14). Drinking-water: Key facts. Retrieved from <https://www.who.int/news-room/fact-sheets/detail/drinking-water>
43. Angulo, F. J., Tippen, T., Sharp, D. J., Payne, B. J., Collier, C., Hill, J. E., Barrett, T. J., Clark, R. M., Geldreich, E. E., Donnell Jr., D., & Swerdlow, D. L. (1997). A community of waterborne outbreak of Salmonellosis and the effectiveness of a boil water order. *American Journal of Public Health, 87*(4), 580-584.
44. Childs, J. W. (2021, February 16). Why Winter Storm Uri caused millions of power outages in Texas. Retrieved from <https://weather.com/news/news/2021-02-16-why-so-many-power-outages-in-texas-winter-storm>
45. Houston Advanced Research Center [HARC]. (2021). Winter Storm Uri’s impact & pathways to resilience in Texas: Power supply impacts. Retrieved from <https://experience.arcgis.com/experience/cc48fcfebfae414b99b3d18f86c72c27/page/page_82/?views=view_6>
46. U.S. Department of Energy [DOE]. (2021, February 21). Extreme cold & winter weather: Update #6 – FINAL. Retrieved from <https://www.energy.gov/sites/prod/files/2021/02/f83/TLP-WHITE_DOE%20Situation%20Update_Cold%20%20Winter%20Weather_%236.pdf>
47. Bandura, A. (1994). Self-efficacy. In V. S. Ramachaudran (Ed.), *Encyclopedia of human behavior* (pp. 71–81). New York: Academic Press.
48. Bandura, A. (1997). *Self-efficacy: The exercise of control.* New York, NY: Freeman. Barnett, D., Thompson, C., Semon, N., Errett, N., Harrison, K., Anderson, M., . . . Storey, J. (2014). EPPM and willingness to respond: The role of risk and efficacy communication in strengthening public health emergency response systems. *Health Communication,* *29*(6), 598-609.
49. Turner, M. M., Rimal, R. N., Morrison, D., & Kim, H. (2006). The role of anxiety in seeking and retaining risk information: Testing the risk perception attitude framework in two studies.*Human Communication Research, 32*(2), 130-156. <https://doi.org/10.1111/j.1468-2958.2006.00006.x>
50. Jerit, J., Zhao, Y., Tan, M., & Wheeler, M. (2018). Differences between national and local media in news coverage of the Zika virus. *Health Communication.* <https://doi.org/10.1080/10410236.2018.1536949>.
51. Day, A. M., O’Shay-Wallace, S., Seeger, M. W., & McElmurry, S. P. (2020). Gender and presence of children: Examining media uses, informational needs, and source preferences during the Flint, Michigan, water crisis. *Journal of International Crisis and Risk Communication Research*, *3*(2). <https://doi.org/10.30658/jicrcr.3.2.2>
52. Day, A. M., O’Shay-Wallace, S., Seeger, M. W., & McElmurry, S. P. (2019). Informational sources, social media use, and race in the Flint, Michigan, water crisis. *Communication Studies, 70*(3), 352-376*.* doi: 10.1080/10510974.2019.1567566
53. Rimal, R. N., Böse, K., Brown, J., Mkandawire, G., & Folda, L. (2009). Extending the purview of the risk perception attitude framework: Findings from HIV/AIDS prevention research in Malawi.*Health Communication, 24*(3), 210-218. <https://doi.org/10.1080/10410230902804109>
54. McDowell, C. P., Andrade, L., Re, V., O’Dwyer, J., Hynds, P. D., & O’Neill, E. (2021). Exploring risk perception and behaviours at the intersection of flood events and private groundwater supplies: A qualitative focus group study.*Water (Basel), 13*(23), 3467. <https://doi.org/10.3390/w13233467>
55. Schuitema, G., Hooks, T., & McDermott, F. (2020). Water quality perceptions and private well management: The role of perceived risks, worry and control.*Journal of Environmental Management, 267.* <https://doi.org/10.1016/j.jenvman.2020.110654>
56. Odimayomi, T. O., Proctor, C. R., Wang, Q. E., Sabbaghi, A., Peterson, K. S., Yu, D. J., Lee, J., Shah, A. D., Ley, C. J., Noh, Y., Smith, C. D., Webster, J. P., Milinkevich, K., Lodewyk, M. W., Jenks, J. A., Smith, J. F., & Whelton, A. J. (2021). Water safety attitudes, risk perception, experiences, and education for households impacted by the 2018 Camp Fire, California.*Natural Hazards (Dordrecht), 108*(1), 947-975. <https://doi.org/10.1007/s11069-021-04714-9>
57. Centers for Disease Control and Prevention. (2022, May 26). Water contamination and diseases. Retrieved from <https://www.cdc.gov/healthywater/drinking/contamination.html?CDC_AA_refVal=https%3A%2F%2Fwww.cdc.gov%2Fhealthywater%2Fdrinking%2Fpublic%2Fwater_diseases.html>
58. Centers for Disease Control and Prevention. (2022, June 30). *Drinking water advisory communication toolbox*. [Retrieved](https://www.cdc.gov/healthywater/emergency/pdf/DWACT-2016.pdf) from <https://www.cdc.gov/healthywater/emergency/dwa-comm-toolbox/index.html>
59. Centers for Disease Control and Prevention. (2021, August 10). Boil water advisory. <https://www.cdc.gov/healthywater/emergency/drinking/drinking-water-advisories/boil-water-advisory.html>
60. Janssen, E., van Osch, L., de Vries, H., & Lechner, L. (2011). Measuring risk perceptions of skin cancer: Reliability and validity of different operationalizations. British Journal of Health Psychology, 16(1), 92-112. <https://doi.org/10.1348/135910710X514120>
61. Islam, K. (2023). Communicating household preparedness for compound public health crisis events: Role of evidence type and crisis message fatigue. <https://proxy.lib.wayne.edu/login?url=https://www.proquest.com/dissertations-theses/communicating-household-preparedness-compound/docview/2871648417/se-2>
62. Tomko, B., Nittrouer, C. L., Sanchez-Vila, X., & Sawyer, A. H. (2023). Disparities in disruptions to public drinking water services in Texas communities during Winter Storm Uri 2021. *PLOS Water, 2*(6), e0000137.
63. Lindell, M. K., Mumpower, J. L., Huang, S. K., Wu, H. C., Samuelson, C. D., & Wei, H. L. (2017). Perceptions of protective actions for a water contamination emergency. *Journal of Risk Research, 20*(7), 887-908.
64. Shafiee, M. E., Berglund, E. Z., & Lindell, M. K. (2018). An agent-based modeling framework for assessing the public health protection of water advisories. *Water Resources Management*, *32*(6), 2033–2059. <https://doi.org/10.1007/s11269-018-1916-6>
65. Lindell, M. K., Mumpower, J. L., Huang, S.-K., Wu, H.-C., Samuelson, C. D., & Wei, H.-L. (2017). Perceptions of protective actions for a water contamination emergency. *Journal of Risk Research*, *20*(7), 887–908. https://doi.org/10.1080/13669877.2015.1121906
66. Wachinger, Renn, O., Begg, C., & Kuhlicke, C. (2013). The risk perception paradox-implications for governance and communication of natural hazards. *Risk Analysis*, *33*(6), 1049–1065. https://doi.org/10.1111/j.1539-6924.2012.01942.x
67. Tomko, B., Nittrouer, C. L., Sanchez-Vila, X., & Sawyer, A. H. (2023). Disparities in disruptions to public drinking water services in Texas communities during Winter Storm Uri 2021. *PLOS Water*, *2*(6), e0000137–. https://doi.org/10.1371/journal.pwat.0000137
68. US Water Alliance. (2019). Closing the water access gap in the United States: A national plan. Retrieved from <https://uswateralliance.org/sites/uswateralliance.org/files/publications/Closing%20the%20Water%20Access%20Gap%20in%20the%20United%20States_DIGITAL.pdf>
69. Nejat, A., Solitare, L., Pettitt, E., & Mohsenian-Rad, H. (2022). Equitable community resilience: The case of Winter Storm Uri in Texas. *International Journal of Disaster Risk Reduction*, *77*, 103070–. https://doi.org/10.1016/j.ijdrr.2022.103070
70. Lee, C-C., Maron, M., & Mostafavi, A. (2022). *Community-scale Big Data Reveals Disparate Impacts of the Texas Winter Storm of 2021 and its Managed Power Outage*. <https://doi.org/10.1057/s41599-022-01353-8>
71. Day, A. M. (2017). Companion animals and natural disasters: A systematic review of literature. *International Journal of Disaster Risk Reduction*, *24*, 81–90. <https://doi.org/10.1016/j.ijdrr.2017.05.015>
72. Day, A. M., & Novak, J. M. (2023). “I couldn't find information for people with pets; so, I gave up,”: Pet owner identity, informational needs, and media uses during Hurricane Harvey. *Western Journal of Communication, 87*(1), 41-64*.* <https://doi.org/10.1080/10570314.2022.2100465>
73. Krumov, K., Schneider, J. F., Jin Liu, Krumova, A. K., Widodo, E., Gungov, A. L., Juhasz, M., Garvanova, M. Z., Kumar, S., & Repaczki, R. (2023). Cross-cultural research of the perceived risk during the COVID-19 pandemic. *Journal of Risk Analysis & Crisis Response (JRACR), 13*(1), 1–18. <https://doi-org.dist.lib.usu.edu/10.54560/jracr.v13i1.352>
74. Kamai, E. M., Calderon, A., Van Horne, Y. O., Bastain, T. M., Breton, C. V., & Johnston, J. E. (2023). Perceptions and experiences of environmental health and risks among Latina mothers in urban Los Angeles, California, USA. *Environmental Health: A Global Access Science Source, 22*(1), 1–13. <https://doi-org.dist.lib.usu.edu/10.1186/s12940-023-00963-2>
75. Girardi, P., Lupo, A., Mastromatteo, L. Y., & Scrimin, S. (2022). Mothers living with contamination of perfluoroalkyl substances: an assessment of the perceived health risk and self-reported diseases. *Environ Sci Pollut Res, 29*(40 pp.60491–60507), 60491–60507. <https://doi-org.dist.lib.usu.edu/10.1007/s11356-022-20085-5>
76. Yalçin, S. S., Gezgen Kesen, G., Güçiz Doğan, B., Yalçin, S., & Acar Vaizoğlu, S. (2023). Mother’s knowledge for environmental risks and self-awareness for the presence of pollutants in her living area in West and Central Anatolia: a cross-sectional survey. *BMC Public Health, 23*(1), 1–15. <https://doi-org.dist.lib.usu.edu/10.1186/s12889-023-16684-7>
77. Griffin, J. S., Hipper, T. J., Chernak, E., Kurapati, P., Lege-Matsuura, J., Popek, L., & Turchi, R. M. (2023). Home-based emergency preparedness for families of children and youth with special healthcare needs: A scoping review. *Health Security, 21*(3), 193–206. <https://doi-org.dist.lib.usu.edu/10.1089/hs.2022.0119>
78. Pescaroli, G., & Alexander, D. (2018). Understanding compound, interconnected, interacting, and cascading risks: A holistic framework. *Risk Analysis*, *38*(11), 2245–2257. <https://doi.org/10.1111/risa.13128>
